# Comparisons of urinary bladder responses to common antimuscarinics reveals unique effects of darifenacin

**DOI:** 10.3389/fphys.2025.1534517

**Published:** 2025-02-20

**Authors:** Vineesha Veer, Russ Chess-Williams, Christian Moro

**Affiliations:** Faculty of Health Sciences and Medicine, Bond University, Gold Coast, Australia

**Keywords:** urinary bladder, bladder pathology, bladder disease, acetylcholine, muscarinic receptor

## Abstract

**Introduction:**

Antimuscarinics are the first-line pharmaceutical treatment for overactive bladder (OAB). However, some literature suggests that responses to these antimuscarinics can influence a variety of non-muscarinic receptors. This study aimed to identify any non-muscarinic influences on contraction from commonly prescribed clinical antimuscarinics using porcine detrusor or urothelium with lamina propria (U&LP) tissues.

**Methods:**

Porcine bladders were dissected into strips of juvenile or adult detrusor or U&LP. Carbachol concentration-response curves were performed on paired tissues in the absence or presence of commonly prescribed antimuscarinics: darifenacin, fesoterodine, oxybutynin, solifenacin, trospium, and tolterodine. Estimated affinities for each antimuscarinic were calculated, and maximum contraction values from control and intervention curves were compared. Experiments in the presence of darifenacin (100 nM) were completed with serotonin (100 µM), prostaglandin E_2_ (10 µM), histamine (100 µM), αβ-methylene-ATP (10 µM), angiotensin II (100 nM), neurokinin A (300 nM), and carbachol (10 µM).

**Results:**

Darifenacin significantly reduced maximum contraction responses to carbachol in adult detrusor preparations by 46%, αβ-methylene-ATP by 50%, prostaglandin E_2_ by 73%, histamine by 64%, and serotonin by 53%. Darifenacin reduced the maximum contraction in adult U&LP preparations to carbachol by 49% and to αβ-methylene-ATP by 35%.

**Discussion:**

Darifenacin presents as an antimuscarinic medication that influences non-muscarinic pathways in urinary bladder tissue, indicating its potential to assist OAB patients with non-muscarinic pathophysiology.

## 1 Introduction

Overactive bladder (OAB) is the most common type of bladder dysfunction, affecting between 11% and 20% of the population and placing heightened burden on healthcare services (Stewart et al., 2003; Chuang et al., 2019). Diagnosis of OAB is symptom-based, with characteristics of urinary urgency, nocturia, and frequency (Haylen et al., 2010). Most bladder contractions that induce urination are due to detrusor M3 receptor activation after acetylcholine release from parasympathetic nerves (Mansfield et al., 2005). However, the urothelium with lamina propria (U&LP) layer also plays a role and can influence detrusor contractions to produce spontaneous contractile activity through the endogenous release of acetylcholine (Moro et al., 2011). Although the underlying cause of OAB is not entirely understood, it is thought that an increased sensitivity to cholinergic activity in the detrusor or an increase in signaling molecules from the U&LP are some of the main involved mechanisms (Brading, 1997; Hanna-Mitchell et al., 2007).

Antimuscarinics, including darifenacin, fesoterodine, oxybutynin, solifenacin, trospium, and tolterodine, are the most commonly prescribed pharmaceutical treatment for OAB (Margulis et al., 2018) with the predominant mechanism of action being a competitive antagonism of M3 receptors in both the detrusor and U&LP of the bladder (Moro et al., 2011; Veer et al., 2023). Although evidence suggests that antimuscarinics alleviate the symptoms of OAB better than placebo formulations (Novara et al., 2008), many OAB patients struggle to maintain their prescribed course of antimuscarinics (Veenboer and Bosch, 2014; Yeowell et al., 2018). In fact, up to 70% of newly diagnosed OAB patients are reported to cease their antimuscarinic treatment regime within the first 12 months of initiation (Vouri et al., 2019). The reason for such a large proportion of OAB patients discontinuing antimuscarinics is not fully understood; however, some evidence suggests a lack of expected benefits or an increase in side effects (Kim et al., 2017).

While most side effects do relate to antimuscarinic actions, some studies suggest that prescribed antimuscarinics possess alternative mechanisms of action (Fetscher et al., 2002), attributing these unintended activated receptors as a possible explanation for the reported side effects and reduced effectiveness. The antimuscarinics oxybutynin, propiverine, solifenacin, and tolterodine have been identified to inhibit non-neuronal ATP release in the human U&LP (Yoshida et al., 2009). A similar mechanism of action for darifenacin was observed in rat urothelium, where both non-neuronal ATP and prostaglandin E_2_ release within this tissue was suppressed by the antimuscarinic (Yokoyama et al., 2011). Although a small subset of studies, these data suggest that antimuscarinics can influence a variety of non-muscarinic receptors across various bladder tissue layers. Furthermore, histamine (Stromberga et al., 2019), neurokinin (Grundy et al., 2018), angiotensin II (Phelps et al., 2022), and 5-hydroxytryptamine (Moro et al., 2016) receptor systems have been shown to influence the contractile activity of the bladder, with the effect of antimuscarinics on these receptors yet to be investigated. This exploratory study aimed to identify any non-muscarinic influences on contraction from commonly prescribed clinical antimuscarinics using detrusor or U&LP tissues.

## 2 Materials and methods

### 2.1 Tissue acquisition and preparation

This exploratory basic study used urinary bladders sourced from female adult sow crossbred Large White Landrace pigs (*Sus scrofa domesticus*, approximately 2 years old, 200 kg live weight) and female juvenile crossbred Large White Landrace pigs (*Sus scrofa domesticus*, approximately 6 months old). Bladders were acquired from the local abattoir after slaughter for the routine commercial provision of food; thus, no animals were bred, harmed, culled, interfered, or interacted with as part of this research project (Queensland Government, 2015). As such, approval from an ethics committee was not required (Queensland Government, 2015). Porcine urinary tissue was used due to its similarity in structure and function to the human bladder (Stromberga et al., 2019; 2020; Phelps et al., 2022).

Bladders were immediately stored and transported to the laboratory after animal slaughter in a 4°C Krebs-bicarbonate solution (“Krebs”): NaCl 118.4 mM, C_6_H_12_O_6_ 11.7 mM, NaHCO_3_ 24.9 mM, KCl 4.6 mM, MgSO_4_ 2.41 mM, KH_2_PO_4_ 1.18 mM, and CaCl_2_ 1.9 mM. In preparation for paired control-experimental tissues, two identical horizontally adjacent strips were dissected from the same anterior wall of the bladder dome region of each bladder. As experiments utilized either the U&LP or detrusor smooth muscle, the U&LP was separated from the detrusor layer using surgical-grade fine scissors, a process that has been established from previous studies (Stromberga et al., 2019; Phelps et al., 2022). During the dissection process, tissues were constantly washed with 4°C Krebs and kept cool. All tissues were dissected to be 1 cm in length and 1 cm in width. Dissected detrusor tissues were measured with an average weight of 225 mg in juvenile (*n* = 140) and 333 mg in adult (*n* = 316) preparations, while U&LP tissue weighed 76 mg in juvenile (*n* = 132) and 110 mg in adult (*n* = 258) preparations. Throughout this manuscript, *n* (number of tissues) values quoted are from paired tissue strips, and as such, the number of animals (N) used can be calculated using *n*÷2.

After dissection, tissues were vertically mounted in isolated tissue baths (Labglass, Brisbane, Australia) with 6 mL Krebs solution at 37°C and gassed with carbogen gas (95% oxygen and 5% carbon dioxide) between a fixed hook and an isometric force transducer to record tension. Both tonic contractions and spontaneous phasic contractions were recorded by a Powerlab system using Labchart v8 (MCT050/D, ADInstruments, Castle Hill, Australia). Tissues were left for 20 min to stabilize to a baseline of 2 g of tension. After stabilization, tissues were then washed three times with warmed fresh Krebs before the addition of any drugs.

### 2.2 Pharmaceutical agents

Commonly prescribed clinical antimuscarinics for OAB were included in concentration-response experiments in female adult (sow) crossbred Large White Landrace porcine samples (Margulis et al., 2018). Optimization of concentrations of clinical antimuscarinics was performed in previous studies using female juvenile (bacon) crossbred Large White Landrace porcine samples (Veer et al., 2023), with concentrations set as darifenacin 100 nM, fesoterodine 100 nM, oxybutynin 1 µM, solifenacin 1 µM, trospium 100 nM, and tolterodine 1 µM. Fesoterodine, oxybutynin, and trospium were dissolved in distilled water. Darifenacin, solifenacin, and tolterodine were dissolved in DMSO. Carbamoylcholine chloride (carbachol) was dissolved in Krebs solution. Dilutions of dissolved antimuscarinics were performed with distilled water. For control experiments, an identical volume of vehicle (DMSO, 0.648 nM) was pipetted into the control tissue bath in the absence of any antimuscarinic. A cumulative concentration-response curve to carbachol was then obtained by increasing carbachol concentrations in half-log unit increments. For darifenacin, to investigate the cause of the observed decrease in maximum contraction, single-concentration experiments to selected agonists known to elicit bladder contractions were performed in both adult and juvenile samples. The following agonists were assessed in the presence and absence of darifenacin (100 nM): serotonin (5HT, 100 µM), prostaglandin E_2_ (PGE2, 10 µM), histamine dihydrochloride (histamine, 100 µM), αβ-methylene ATP (αβm-ATP, 10 µM), angiotensin II (ATII, 100 nM), neurokinin A (NKA, 300 nM), and carbachol (10 µM) with concentrations justified and aligning with previous studies (Phelps et al., 2022; Veer et al., 2023).

Further studies were conducted on non-muscarinic agonists that were identified to be influenced by darifenacin. To assess whether darifenacin’s influence on non-muscarinic agonists is mediated through a muscarinic receptor-related pathway or an altogether separate pathway, studies with atropine (1 µM), 5HT (100 µM), PGE2 (10 µM), histamine (100 µM), and αβm-ATP (10 µM) were undertaken in the presence and absence of darifenacin (100 nM). PGE2 was dissolved in 100% ethanol, and all other agonists and atropine were dissolved in and diluted with distilled water. All antimuscarinic drugs and non-muscarinic agonists were sourced from Sapphire Bioscience (Redfern, Australia), and carbachol and atropine were sourced from Sigma-Aldrich (St Louis, United States).

### 2.3 Data retrieval and measurements

A 20-min treatment of the intervention tissue with an antimuscarinic in the isolated tissue bath was completed, with the average baseline tension recorded before the addition of any agonist across all studies. For concentration-response experiments, tension was recorded at the peak level of contraction after each cumulative dose of carbachol. For single-concentration experiments, the peak contractile response of the tissue to the agonist was recorded. At the end of every experiment, individual tissues were weighed in milligrams. Two paired tissues were taken from each animal. As such, the stated value of *n* represents the number of paired tissue samples. A prespecified minimum of *n* = 8 paired tissues was determined for experiments conducted for each drug and tissue type beforehand. However, in cases where additional tissues were available for collection from the abattoir, additional experiments could be run up to *n* = 11.

### 2.4 Data analysis

Data were analyzed and graphed using GraphPad Prism v9.2 (GraphPad Software, La Jolla, United States). Normality tests were conducted on all data and presented enough evidence to accept the null hypothesis of a normal distribution (D'Agostino–Pearson test, *alpha* >0.05). Analyses used were specific to each study. Analysis for concentration-response experiments included the extraction of pEC50s with standard deviation (SD) to determine changes in carbachol potency between the control and intervention groups with each antimuscarinic (paired Student’s two-tailed *t*-test), and *p <* 0.05 was considered significant. An assessment was also made between the maximum contraction for the control and intervention groups in response to each antimuscarinic (paired Student’s two-tailed *t*-test). The estimated affinity (pKD) was calculated from the shift of the carbachol curves and reported as mean ± SD. For single-concentration response studies, the change in tension was calculated for all tissues, and comparisons of the tension were completed between the control and intervention tissues (paired Student’s two-tailed *t*-test). Where data were normalized, 100% was designated for the point of maximum contraction of the tissue, and 0% was designated as the initial resting tone prior to initial contraction.

## 3 Results

### 3.1 Clinical antimuscarinics influence on detrusor and U&LP contractions

**FIGURE 1:**
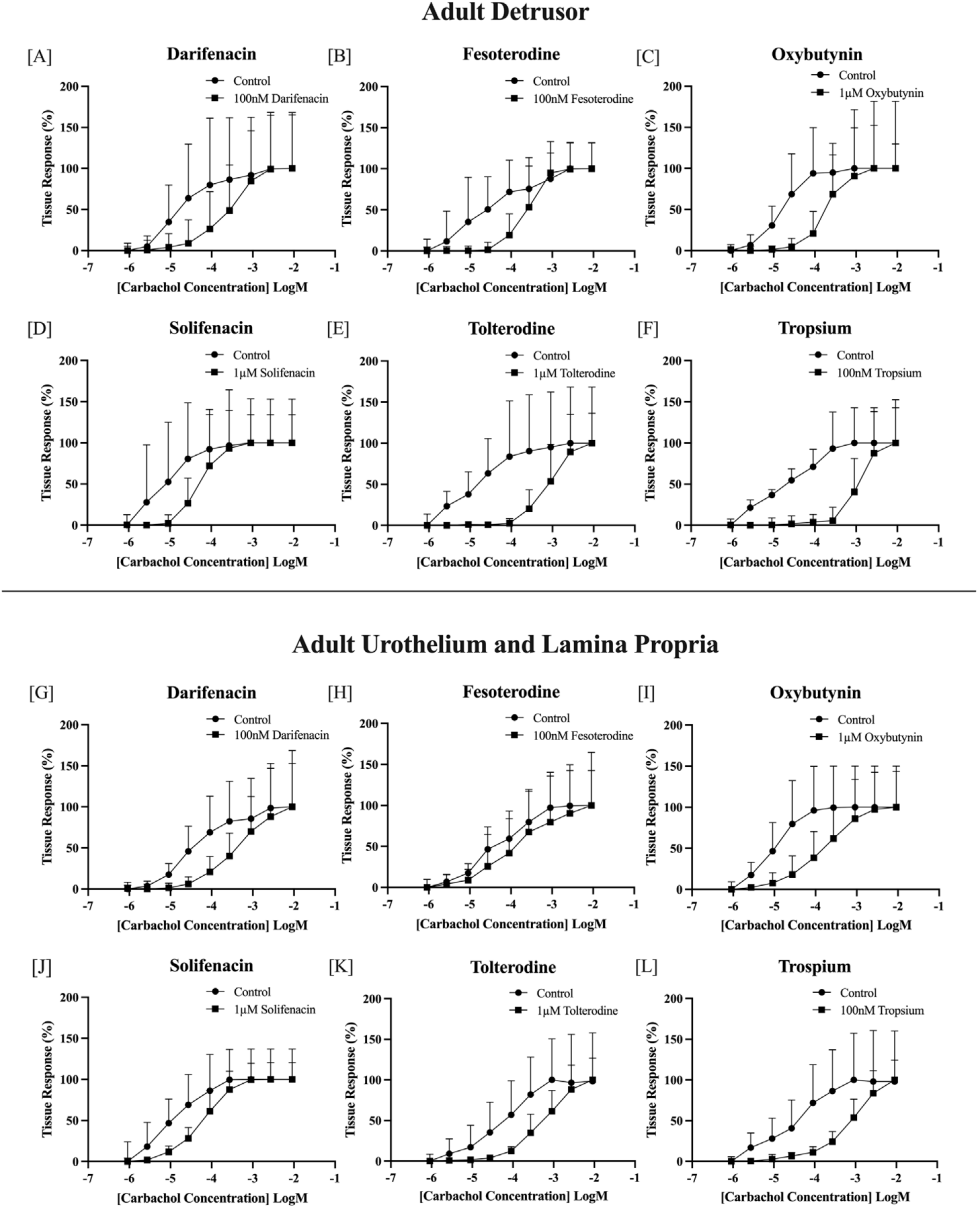
Normalized carbachol concentration-response curves of adult bladder detrusor in the presence and absence of: darifenacin [100 nM, *n* = 9, **(A)**]; fesoterodine [100 nM, *n* = 8, **(B)**]; oxybutynin [1 μM, *n* = 9, **(C)**]; solifenacin [1 μM, *n* = 8, **(D)**]; tolterodine [1 μM, *n* = 8, **(E)**]; and trospium [100 nM, *n* = 9, **(F)**]. Normalized carbachol concentration-response curves of adult bladder U&LP in the presence and absence of darifenacin [100 nM, *n* = 9, **(G)**]; fesoterodine [100 nM, *n* = 8, **(H)**]; oxybutynin [1 μM, *n* = 11, **(I)**]; solifenacin [1μM, *n* = 8, **(J)**]; tolterodine [1 μM, *n* = 8, **(K)**]; and trospium [100 nM, *n* = 8, **(L)**]. U&LP = Urothelium with lamina propria. Data points are represented as the percentage of the maximum contraction of the tissue ± SD.

Carbachol concentration-response curves were successfully obtained, with all antimuscarinics producing significant parallel shifts to the right (Figure 1) when compared to their paired control tissue (*p <* 0.05 for all, paired Student’s two-tailed *t*-test), with the estimated pKD values calculated for both the detrusor and U&LP (represented as mean ± SD). The antimuscarinic exhibiting the highest estimated pKD in the detrusor was trospium (9.30 ± 1.07, *n* = 9), followed by fesoterodine (8.55 ± 1.02, *n* = 8), darifenacin (7.95 ± 1.19, *n* = 9), tolterodine (7.93 ± 0.94, *n* = 8), oxybutynin (7.44 ± 1.00, *n* = 9), and solifenacin (6.63 ± 0.77, *n* = 8). The antimuscarinic exhibiting the highest estimated pKD in the U&LP was trospium (8.46 ± 0.43, *n* = 8), followed by darifenacin (7.97 ± 0.54, *n* = 9), fesoterodine (7.95 ± 1.30, *n* = 8), tolterodine (7.58 ± 0.44, *n* = 8), oxybutynin (7.32 ± 0.70, *n* = 11), and lowest being solifenacin (6.96 ± 0.63, *n* = 8).

### 3.2 Investigating darifenacin’s influence on reducing maximum contraction

Darifenacin was unique among the other antagonists tested as it not only shifted the curve to the right but also significantly depressed the maximum contractile force exhibited by carbachol in both adult detrusor and U&LP.

For adult samples treated with darifenacin during cumulative concentration-response curves, a 34% reduction in maximum contraction occurred when compared to their respective paired control strips in the detrusor (100 nM, *n* = 9, *p <* 0.01, Table 1). In adult U&LP, a 32% reduction in maximum contraction occurred (100 nM, *n* = 9, *p <* 0.05) between control tissues and those treated with darifenacin.

**TABLE 1:** Detrusor and U&LP tissue maximum contractions in adult and juvenile samples (mN) in response to increasing increments of carbachol (910 nM–9.1075 mM) in the absence and presence of 100 nM darifenacin (mean ± SD), with comparisons between the paired intervention and control group for each tissue (*p < 0.05, **p < 0.01, paired two-tailed Student’s t-test).

Tissue	Detrusor			U&LP		
	Absence (mN)	Presence (mN)	*n*	Absence (mN)	Presence (mN)	*n*
Adult	19.89 ± 12.73	12.05 ± 8.35**	9	22.93 ± 11.50	14.49 ± 10.10*	9
Juvenile	57.89 ± 33.65	22.39 ± 9.55**	11	46.11 ± 17.96	29.85 ± 13.97*	10

For these juvenile samples, when treated with darifenacin, a 41% reduction in maximum contraction was identified in the detrusor (100 nM, *n* = 11, *p* < 0.01, Table 1). In juvenile U&LP, a 21% reduction in maximum contraction occurred (100 nM, *n* = 10, *p* < 0.05).

A comparison between adult and juvenile and detrusor and U&LP tissues was performed to assess the magnitude of darifenacin’s influence toward inhibiting the maximum contraction to carbachol. There were no significant differences between the magnitude of darifenacin inhibition in either adult or juvenile tissues within each layer (*p* = NSD).

### 3.3 Single-concentration applications of agonists in the presence of darifenacin in the adult model

To further assess the influence of darifenacin on maximum contraction, additional experiments were set up where a single concentration of agonist was added in the absence and presence of the antimuscarinic. This would investigate the influence of darifenacin on other prominent contractile-mediating receptors within the urinary bladder, including purinergic, prostaglandin, histamine, 5-HT, neurokinin, and angiotensin. The single-concentration setup was used as not all agonists would produce concentration-response curves due to receptor desensitization, limited selectivity, or a minimal range of effective concentrations.

**FIGURE 2:**
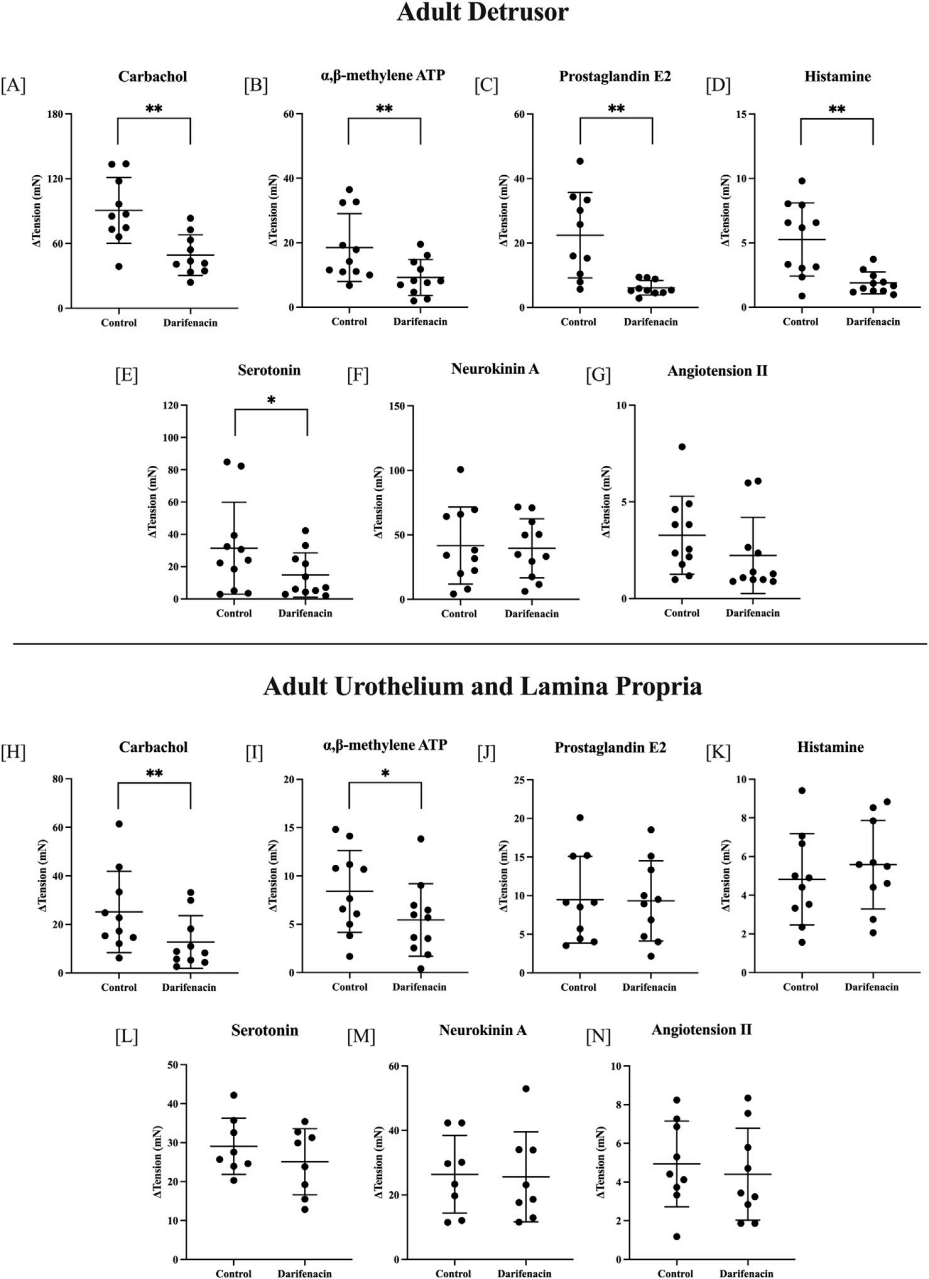
Single-concentration applications of various agonists in adult tissues. Detrusor changes (ΔmN ± SD) in baseline tension in the presence and absence of darifenacin for carbachol [10 µM, *n* = 10, **(A)**]; αβm-ATP [10 µM, *n* = 11, **(B)**]; PGE2 [10 µM, *n* = 10, **(C)**]; histamine [100 µM, *n* = 11, **(D)**]; 5HT [100 µM, *n* = 11, **(E)**]; NKA [300 nM, *n* = 11, **(F)**]; and ATII [100 nM, *n* = 11, **(G)**]. U&LP changes in baseline tension in the presence and absence of darifenacin for carbachol [10 µM, n = 10, **(H)**]; αβm-ATP [10 µM, *n* = 11, **(I)**]; PGE2 [10 µM, *n* = 10, **(J)**]; histamine [100 µM, *n* = 10, **(K)**]; 5HT [100 µM, *n* = 10, **(L)**]; NKA [300 nM, *n* = 8, **(M)**]; and ATII [100 nM, *n* = 10, **(N)**]. *p < 0.05, **p < 0.01, paired two-tailed Student’s t-test.

In adult detrusor preparations, darifenacin (100 nM) significantly decreased the contractile response to carbachol by 41.48 ± 37.81 mN (46%, 10 µM, *n* = 10, *p <* 0.01), to αβm-ATP by 9.25 ± 7.78 mN (50%, 10 µM, *n* = 11, *p <* 0.01), to PGE2 by 16.30 ± 12.56 mN (73%, 10 µM, *n* = 10, *p <* 0.01), to histamine by 3.37 ± 2.89 mN (64%, 100 µM, *n* = 11, *p <* 0.01), and to 5HT by 16.62 ± 21.28 mN (53%, 100 µM, *n* = 11, *p <* 0.05, Figure 2). Adult detrusor responses to NKA and AII were not influenced by the presence of darifenacin. In U&LP preparations, darifenacin significantly decreased the contractile response to carbachol by 12.42 ± 7.24 mN (49%, 10 µM, *n* = 10, *p <* 0.01) and to αβm-ATP by 2.95 ± 4.16 mN (35%, 10 µM, *n* = 11, *p <* 0.05, Figure 2). All other agonists were not influenced by the presence of darifenacin in the adult U&LP tissue.

To remove the influence of muscarinic receptor stimulation, a series of experiments were conducted in the presence of atropine. In the presence of only atropine (1 µM) or both atropine (1 µM) and darifenacin (100 nM), adult detrusor tissues were stimulated with αβm-ATP (*n* = 8, 10 µM), PGE2 (*n* = 8, 10 µM), and histamine (*n* = 8, 100 µM), 5HT (*n* = 8, 100 µM), and adult U&LP tissues were stimulated with the presence of αβm-ATP (*n* = 8, 10 µM). There was no difference between responses for tissues in the presence of solely atropine or atropine and darifenacin (*p* = NSD).

### 3.4 Influence of darifenacin on the detrusor and U&LP in the juvenile model

**FIGURE 3:**
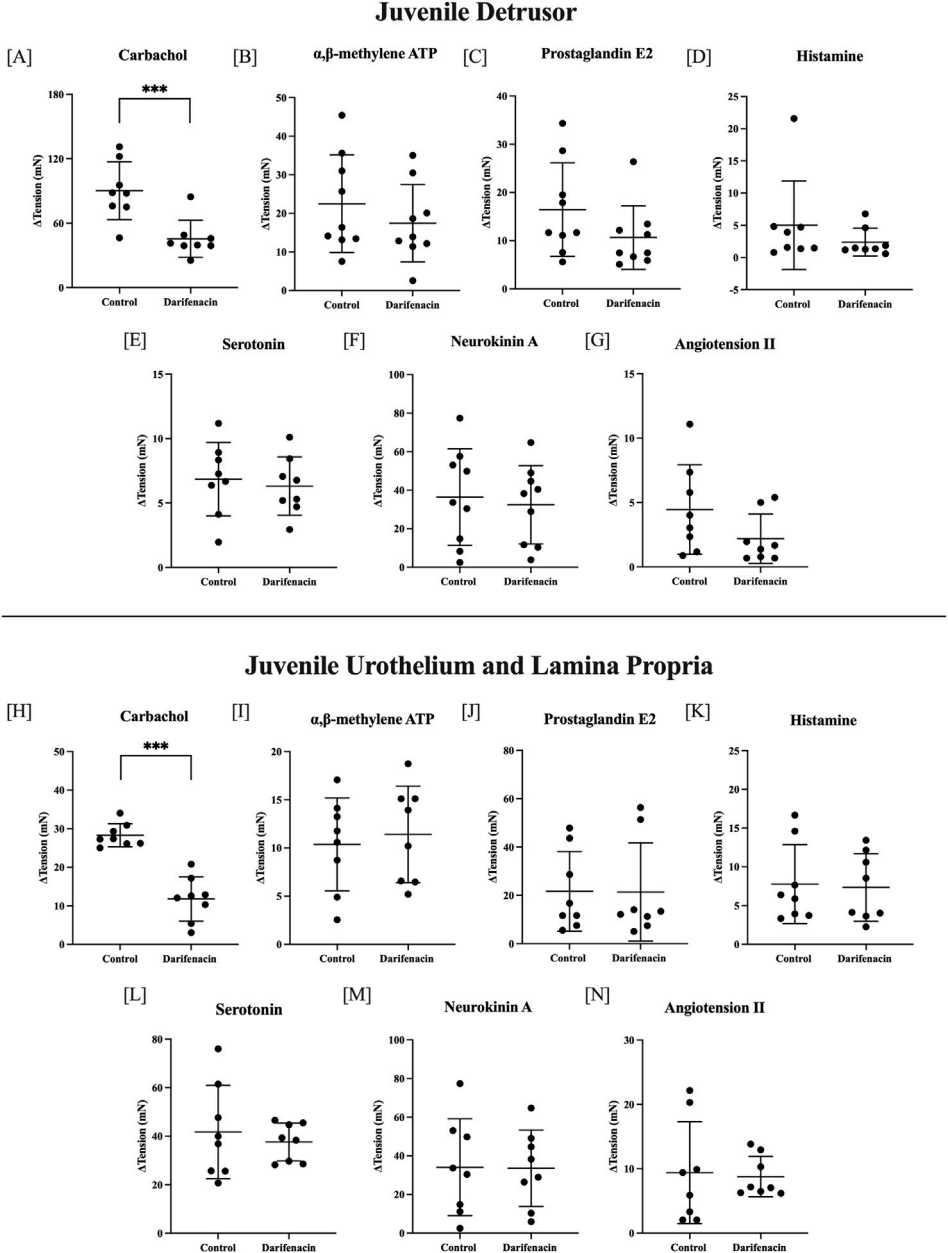
Single-concentration applications of various agonists in juvenile tissues. Detrusor changes (ΔmN ± SD) in baseline tension in the presence and absence of darifenacin for carbachol [10 µM, n = 8, **(A)**]; αβm-ATP [10 µM, *n* = 9, **(B)**]; PGE2 [10 µM, *n* = 9, **(C)**]; histamine [100 µM, *n* = 8, **(D)**]; 5HT [100 µM, *n* = 8, **(E)**], NKA [300 nM, *n* = 9, **(F)**]; and ATII [100 nM, *n* = 8, **(G)**]. U&LP changes in baseline tension in the presence and absence of darifenacin for carbachol [10 µM, n = 8 **(H)**]; αβm-ATP [10 µM, *n* = 8, **(I)**]; PGE2 [10 µM, *n* = 8, **(J)**]; histamine [100 µM, *n* = 8, **(K)**]; 5HT [100 µM, *n* = 8, **(L)**]; NKA [300 nM, *n* = 8, **(M)**]; and ATII [100 nM, *n* = 8, **(N)**]. Comparisons between the paired control and intervention tissues were made (*p < 0.05, **p < 0.01, ***p < 0.001, paired two-tailed Student’s *t*-test).

In juvenile tissues, the effect of darifenacin was examined in seven single-concentration experiments. In detrusor preparations, darifenacin significantly decreased the contractile response to carbachol by 44.87 ± 19.43 mN (50%, 10 µM, *n* = 8, *p <* 0.001). No other agonists influenced detrusor tissue contractions in the presence of darifenacin. In U&LP preparations, darifenacin significantly decreased the contractile response to carbachol by 16.50 ± 5.82 mN (58%, 10 µM, *n* = 8, *p <* 0.001, Figure 3). No other agonists influenced U&LP tissue contractions in the presence of darifenacin.

## 4 Discussion

The identified low rates of adherence with OAB treatment regimens have been an ongoing issue, and there is value in furthering the understanding of how this class of medications impacts various receptors within the urinary bladder. Application of all antimuscarinics inhibited carbachol-induced contractions in both the adult U&LP and detrusor tissues. Our previous study Veer et al., (2023) includes published carbachol-induced concentration curves using juvenile urinary bladders in the presence of antimuscarinics. As such, there was interest in assessing the potential for this antimuscarinic to inhibit maximum contraction on both juvenile and adult tissues within the present study. When comparing the tissue response to maximum contraction for oxybutynin, solifenacin, fesoterodine, tolterodine, and trospium to their respective control tissue, these antimuscarinics acted within expectations. This study reports a considerable impact of darifenacin, where a decrease in the tissue’s maximum contraction was observed.

Darifenacin’s action as an insurmountable antagonist has been suggested in previous literature using human (Fetscher et al., 2002), canine (Choppin and Eglen, 2001), and rat (Hegde et al., 1997; Kories et al., 2003) bladders. In the current study, darifenacin-induced inhibition to maximum contraction was observed across U&LP and detrusor layers in both adult and juvenile tissues. While the alternative mechanisms of darifenacin in adult tissue were identified, the reason for the depressed maximum contraction in juvenile detrusor and U&LP tissue is unknown. The lack of recognized alternative receptor targets in juvenile tissue suggests this might be from an influence on second messenger pathways, such as extracellular Ca^2+^ through L-type channels, intracellular Ca^2+^ from the sarcoplasmic reticulum, or Rho kinase pathways (Phelps et al., 2022; 2023).

While single-concentration experiments are not able to differentiate between potency and maximum effect, the potential for darifenacin to exhibit a muscarinic receptor-mediated influence was eliminated in this study by a lack of any difference in the presence of atropine for αβm-ATP, PGE2, histamine, or 5HT. It is still unclear what the pathway of this mechanism is and whether there are actions on the tissue itself or if the innervating neurons are activated to release neurotransmitters (including acetylcholine). However, other antimuscarinics, such as propiverine, are well known to inhibit voltage-operated calcium channels found in detrusor muscle cells (Wuest et al., 2005). Oxybutynin and solifenacin have also been identified to possess a weak Ca^2+^ antagonistic effect, though it is thought to be clinically irrelevant because this mechanism only occurs at a high concentration (Yono et al., 1999; Masunaga et al., 2008). These results potentially suggest that darifenacin may even be acting directly on the voltage-gated channels, providing a reason for the depressed maximum contraction. Past research by Miyamae et al. (2003) using 80 mmol/L KCl on human detrusor tissue found that there was no change in contractions after the addition of darifenacin. However, it would be of great interest for future research if these experiments were repeated on porcine tissue or if additional studies could be performed that isolate and target calcium receptors to further assess this as a probable mechanism.

Clinical studies focused on human patient compliance with darifenacin report that adherence increases with age (Wagg et al., 2012; Mauseth et al., 2013). While direct action on muscarinic receptors is the prominent action of darifenacin, its influence on other receptors in aged tissue might be of great interest. Strong bladder contractions have been identified in response to purinergic (Kumar et al., 2010), serotonin (Moro et al., 2016), histamine (Stromberga et al., 2019), and prostaglandin (Stromberga et al., 2020). This means that dysfunction of these receptor systems, or secretion of their ligands around the smooth muscle, may induce contractions that are sensitive to antagonism by darifenacin.

Limitations of this study include the use of single-concentration responses for non-muscarinic agonists. Although our study identifies the influence of darifenacin on these six agonists, assessing the influence of darifenacin on other ion channels or second messenger pathways in the bladder could provide further insight into its mechanisms. As the focus of the study was antimuscarinic inhibitions to maximum contraction, future studies could assess any enhancements to maximum contractile activity as well. An insightful future study would be to further assess the mechanism of darifenacin and whether its antagonism occurs through calcium channel blockage.

## 5 Conclusion

With the current challenges of patient compliance to antimuscarinics for the treatment of OAB, this study investigated the effects of antimuscarinic medications on the different layers of the bladder. Darifenacin emerged as an antimuscarinic with alternative targets in the bladder, suppressing PGE2, αβm-ATP, 5HT, histamine, and muscarinic-induced contractions in the detrusor, and αβm-ATP and muscarinic-induced contractions in the U&LP. Further suggesting that darifenacin has a non-muscarinic action which might contribute to its therapeutic effect.

## Data Availability

The raw data supporting the conclusions of this article will be made available by the authors, without undue reservation.

## References

[B1] Brading A. F. (1997). A myogenic basis for the overactive bladder. Urology 50 (6A Suppl. l), 57–73. 10.1016/s0090-4295(97)00591-8 9426752

[B2] Choppin A. Eglen R. M. (2001). Pharmacological characterization of muscarinic receptors in dog isolated ciliary and urinary bladder smooth muscle. Br. J. Pharmacol. 132 (4), 835–842. 10.1038/sj.bjp.0703901 11181424 PMC1572633

[B3] Chuang Y.-C. Liu S.-P. Lee K.-S. Liao L. Wang J. Yoo T. K. (2019). Prevalence of overactive bladder in China, Taiwan and South Korea: results from a cross-sectional, population-based study. Low. Urin. Tract. Symptoms 11 (1), 48–55. 10.1111/luts.12193 28967230 PMC7379992

[B4] Fetscher C. Fleichman M. Schmidt M. Krege S. Michel M. C. (2002). M(3) muscarinic receptors mediate contraction of human urinary bladder. Br. J. Pharmacol. 136 (5), 641–643. 10.1038/sj.bjp.0704781 12086973 PMC1573406

[B5] Grundy L. Chess-Williams R. Brierley S. M. Mills K. Moore K. H. Mansfield K. (2018). NKA enhances bladder-afferent mechanosensitivity via urothelial and detrusor activation. Am. J. Physiology - Ren. Physiology 315 (4), F1174–f1185. 10.1152/ajprenal.00106.2018 PMC6230738 29897284

[B6] Hanna-Mitchell A. T. Beckel J. M. Barbadora S. Kanai A. J. de Groat W. C. Birder L. A. (2007). Non-neuronal acetylcholine and urinary bladder urothelium. Life Sci. 80 (24-25), 2298–2302. 10.1016/j.lfs.2007.02.010 17363007 PMC3085916

[B7] Haylen B. T. de Ridder D. Freeman R. M. Swift S. E. Berghmans B. Lee J. (2010). An international urogynecological association (IUGA)/international continence society (ICS) joint report on the terminology for female pelvic floor dysfunction. Neurourol. Urodynamics 29 (1), 4–20. 10.1002/nau.20798 19941278

[B8] Hegde S. S. Choppin A. Bonhaus D. Briaud S. Loeb M. Moy T. M. (1997). Functional role of M2 and M3 muscarinic receptors in the urinary bladder of rats *in vitro* and *in vivo* . Br. J. Pharmacol. 120 (8), 1409–1418. 10.1038/sj.bjp.0701048 9113359 PMC1564615

[B9] Kim A. Lee K. S. Jung R. Na S. Kim J. C. Kim H. G. (2017). Health related quality of life in patients with side-effects after antimuscarinic treatment for overactive bladder. Low. Urin. Tract. Symptoms 9 (3), 171–175. 10.1111/luts.12132 27291463

[B10] Kories C. Czyborra C. Fetscher C. Schneider T. Krege S. Michel M. C. (2003). Gender comparison of muscarinic receptor expression and function in rat and human urinary bladder: differential regulation of M2 and M3 receptors? Naunyn-Schmiedeberg's Archives Pharmacol. 367 (5), 524–531. 10.1007/s00210-003-0713-8 12669188

[B11] Kumar V. Chapple C. R. Rosario D. Tophill P. R. Chess-Williams R. (2010). *In vitro* release of adenosine triphosphate from the urothelium of human bladders with detrusor overactivity, both neurogenic and idiopathic. Eur. Urol. 57 (6), 1087–1092. 10.1016/j.eururo.2009.11.042 20022422

[B12] Mansfield K. J. Liu L. Mitchelson F. J. Moore K. H. Millard R. J. Burcher E. (2005). Muscarinic receptor subtypes in human bladder detrusor and mucosa, studied by radioligand binding and quantitative competitive RT-PCR: changes in ageing. Br. J. Pharmacol. 144 (8), 1089–1099. 10.1038/sj.bjp.0706147 15723094 PMC1576093

[B13] Margulis A. V. Linder M. Arana A. Pottegård A. Berglind I. A. Bui C. L. (2018). Patterns of use of antimuscarinic drugs to treat overactive bladder in Denmark, Sweden, and the United Kingdom. PloS one 13 (9), e0204456. 10.1371/journal.pone.0204456 30260993 PMC6160033

[B14] Masunaga K. Yoshida M. Inadome A. Murakami S. Sugiyama Y. Satoji Y. (2008). Pharmacological effects of solifenacin on human isolated urinary bladder. Pharmacology 82 (1), 43–52. 10.1159/000127840 18434763

[B15] Mauseth S. A. Skurtveit S. Spigset O. (2013). Adherence, persistence and switch rates for anticholinergic drugs used for overactive bladder in women: data from the Norwegian Prescription Database. Acta Obstetricia Gynecol. Scand. 92 (10), 1208–1215. 10.1111/aogs.12196 23763552

[B16] Miyamae K. Yoshida M. Murakami S. Iwashita H. Ohtani M. Masunaga K. (2003). Pharmacological effects of darifenacin on human isolated urinary bladder. Pharmacology 69 (4), 205–211. 10.1159/000073665 14624061

[B17] Moro C. Edwards L. Chess-Williams R. (2016). 5-HT2A receptor enhancement of contractile activity of the porcine urothelium and lamina propria. Int. J. Urology 23 (11), 946–951. 10.1111/iju.13172 27531585

[B18] Moro C. Uchiyama J. Chess-Williams R. (2011). Urothelial/lamina propria spontaneous activity and the role of M3 muscarinic receptors in mediating rate responses to stretch and carbachol. Urology 78 (6), 1442.e9–1442.e1.442E15. 10.1016/j.urology.2011.08.039 22001099

[B19] Novara G. Galfano A. Secco S. D'Elia C. Cavalleri S. Ficarra V. (2008). A systematic review and meta-analysis of randomized controlled trials with antimuscarinic drugs for overactive bladder. Eur. Urol. 54 (4), 740–763. 10.1016/j.eururo.2008.06.080 18632201

[B20] Phelps C. Chess-Williams R. Moro C. (2022). The dependence of urinary bladder responses on extracellular calcium varies between muscarinic, histamine, 5-HT (serotonin), neurokinin, prostaglandin, and angiotensin receptor activation. Front. Physiology 13, 841181. 10.3389/fphys.2022.841181 PMC9008219 35431993

[B21] Phelps C. Chess-Williams R. Moro C. (2023). The role of intracellular calcium and Rho kinase pathways in G protein-coupled receptor-mediated contractions of urinary bladder urothelium and lamina propria. Am. J. Physiology-Cell Physiology 324, C787–C797. 10.1152/ajpcell.00441.2022 PMC10027080 36689673

[B22] Queensland Government (2015). Using animals for scientific purposes. Available at: https://www.business.qld.gov.au/industries/farms-fishing-forestry/agriculture/livestock/animal-welfare/animals-science/using-animals (Accessed October 10, 2022).

[B23] Stewart W. F. Van Rooyen J. B. Cundiff G. W. Abrams P. Herzog A. R. Corey R. (2003). Prevalence and burden of overactive bladder in the United States. World J. Urology 20 (6), 327–336. 10.1007/s00345-002-0301-4 12811491

[B24] Stromberga Z. Chess-Williams R. Moro C. (2019). Histamine modulation of urinary bladder urothelium, lamina propria and detrusor contractile activity via H1 and H2 receptors. Sci. Rep. 9 (1), 3899. 10.1038/s41598-019-40384-1 30846750 PMC6405771

[B25] Stromberga Z. Chess-Williams R. Moro C. (2020). The five primary prostaglandins stimulate contractions and phasic activity of the urinary bladder urothelium, lamina propria and detrusor. BMC Urol. 20 (1), 48. 10.1186/s12894-020-00619-0 32349725 PMC7191740

[B26] Veenboer P. W. Bosch J. L. H. R. (2014). Long-term adherence to antimuscarinic therapy in everyday practice: a systematic review. J. Urology 191 (4), 1003–1008. 10.1016/j.juro.2013.10.046 24140548

[B27] Veer V. Chess-William R. Moro C. (2023). Antimuscarinic actions on bladder urothelium and lamina propria contractions are similar to those observed in detrusor smooth muscle preparations. Neurourol. Urodynamics 42, 1080–1087. 10.1002/nau.25176 36975140

[B28] Vouri S. M. Schootman M. Strope S. A. Xian H. Olsen M. A. (2019). Antimuscarinic use and discontinuation in an older adult population. Archives Gerontology Geriatrics 80, 1–11. 10.1016/j.archger.2018.09.005 PMC6497475 30268971

[B29] Wagg A. Compion G. Fahey A. Siddiqui E. (2012). Persistence with prescribed antimuscarinic therapy for overactive bladder: a UK experience. BJU Int. 110 (11), 1767–1774. 10.1111/j.1464-410X.2012.11023.x 22409769

[B30] Wuest M. Hecht J. Christ T. Braeter M. Schoeberl C. Hakenberg O. W. (2005). Pharmacodynamics of propiverine and three of its main metabolites on detrusor contraction. Br. J. Pharmacol. 145 (5), 608–619. 10.1038/sj.bjp.0706244 15880140 PMC1576185

[B31] Yeowell G. Smith P. Nazir J. Hakimi Z. Siddiqui E. Fatoye F. (2018). Real-world persistence and adherence to oral antimuscarinics and mirabegron in patients with overactive bladder (OAB): a systematic literature review. Br. Med. J. Open 8 (11), e021889. 10.1136/bmjopen-2018-021889 PMC6252764 30467131

[B32] Yokoyama O. Tanaka I. Kusukawa N. Yamauchi H. Ito H. Aoki Y. (2011). Antimuscarinics suppress adenosine triphosphate and prostaglandin E2 release from urothelium with potential improvement in detrusor overactivity in rats with cerebral infarction. J. Urology 185 (6), 2392–2397. 10.1016/j.juro.2011.02.048 21511278

[B33] Yono M. Yoshida M. Wada Y. Kikukawa H. Takahashi W. Inadome A. (1999). Pharmacological effects of tolterodine on human isolated urinary bladder. Eur. J. Pharmacol. 368 (2-3), 223–230. 10.1016/s0014-2999(99)00036-9 10193658

[B34] Yoshida M. Masunaga K. Nagata T. Maeda Y. Miyamoto Y. Kudoh J. (2009). Attenuation of non-neuronal adenosin triphosphate release from human bladder mucosa by antimuscarinic agents. Low. Urin. Tract. Symptoms 1 (2), 88–92. 10.1111/j.1757-5672.2009.00049.x

